# The Relevance of *HLA* Sequencing in Population Genetics Studies

**DOI:** 10.1155/2014/971818

**Published:** 2014-07-15

**Authors:** Alicia Sanchez-Mazas, Diogo Meyer

**Affiliations:** ^1^Department of Genetics and Evolution—Anthropology Unit, University of Geneva and Institute of Genetics and Genomics of Geneva (IGE3), 12 Rue Gustave-Revilliod, 1211 Geneva 4, Switzerland; ^2^Department of Genetics and Evolutionary Biology, University of São Paulo, Rua do Matão 277, São Paulo, SP 05508-090, Brazil

## Abstract

Next generation sequencing (NGS) is currently being adapted by different biotechnological platforms to the standard typing method for *HLA* polymorphism, the huge diversity of which makes this initiative particularly challenging. Boosting the molecular characterization of the *HLA* genes through efficient, rapid, and low-cost technologies is expected to amplify the success of tissue transplantation by enabling us to find donor-recipient matching for rare phenotypes. But the application of NGS technologies to the molecular mapping of the MHC region also anticipates essential changes in population genetic studies. Huge amounts of *HLA* sequence data will be available in the next years for different populations, with the potential to change our understanding of *HLA* variation in humans. In this review, we first explain how *HLA* sequencing allows a better assessment of the *HLA* diversity in human populations, taking also into account the methodological difficulties it introduces at the statistical level; secondly, we show how analyzing *HLA* sequence variation may improve our comprehension of population genetic relationships by facilitating the identification of demographic events that marked human evolution; finally, we discuss the interest of both *HLA* and genome-wide sequencing and genotyping in detecting functionally significant SNPs in the MHC region, the latter having also contributed to the makeup of the *HLA* molecular diversity observed today.

## 1. Introduction

About four decades ago, when* HLA*-typed population samples from different geographic locations started to be available for comparative analyses, both allele frequencies and linkage disequilibrium measures revealed extensive geographic variation (e.g., [1–4] for Europe). These patterns were interpreted as explicit signals of human migration history, based on which impressive syntheses on “classical” polymorphic markers were published [5]. At the time of these studies, however, a very limited part of the* HLA* polymorphism was described as it merely consisted of a few dozens of alleles at three main genes (A, B, and DRB1). As the number of known alleles increased thanks to the development of powerful molecular technology, to a growing interest in anthropology within the frame of international histocompatibility and immunogenetics workshops [6–11], and to continuous efforts of transplantation laboratories to type potential bone marrow donors, the overall picture became more complex: above all, distinctive sets of alleles, as well as unusual frequencies of a “blank” (i.e., the sum of frequencies of putative unknown alleles), were observed in different populations, raising new challenges to the estimation of genetic distances and other useful statistics. In parallel, an important role of natural selection in shaping the patterns of* HLA* diversity became visible in the form of both a significant excess of heterozygosity in most populations and an excess of nonsynonymous polymorphism within* HLA* loci. Such results provided evidence that the immunological function within* HLA* genes was a target of balancing selection (see [12] for a review). An increasing number of associations, both positive and negative, between* HLA* alleles and diseases were also proposed (reviewed in [13]), the results being often heterogeneous across studies and/or populations. Last but not least, the finding of interactions between* HLA* and other highly polymorphic molecules of the immune system, such as KIR, raised the possibility of immune-mediated coevolution between* HLA* and other gene families [14].

As described above, in the last decades not only has the* HLA* polymorphism become very hard to describe in human populations, due to its molecular complexity, but also its interpretation turned out to be much more challenging in view of the intricate mechanisms driving its evolution. Fortunately, the development of constantly updated repositories of allele definitions [15], of powerful biostatistical tools taking into account typing ambiguities to analyze genetic diversity [10, 16], and of large databases of* HLA* frequencies in populations from all over the world [17–19] simplified the analysis of these data. As a result, new studies confirmed the important role played by geography and demographic history in the evolution of the* HLA* polymorphism in modern humans, for example, in Europe [20], and allowed better assessments of natural selection acting on the different* HLA* genes [19, 21–24]. On the other hand, these works are still based on a partial description of the molecular variation in the MHC region.

Now next generation sequencing (NGS) may fill this gap. Although this technology is not yet applied to* HLA* in routine practice due to methodological, mainly bioinformatics, complications [25], large amounts of* HLA* sequence data will be available in the next few years for population genetic studies. In this review, we first explain how using DNA sequence data will improve the assessment of* HLA* population diversity compared to the present situation; we then show how different methods adapted to these data will help in reconstructing the evolution of modern humans taking into account both demography and natural selection. Finally, we discuss how both* HLA* and genome-wide sequencing will lead to the detection of functionally important polymorphic sites in the MHC region, with crucial implications in population genetics. Overall, these approaches will provide a better comprehension of the molecular evolution of this amazing polymorphism.

## 2. Improving the Assessment of Population Diversity through* HLA* Sequencing

The genetic profile of a given population is generally described by a number of observed alleles to which relative frequencies are assigned. Such a distribution is currently inferred from a list of possible genotypes deduced from the molecular typing of a sample of individuals representative of this population. However, so far the* HLA* typing techniques most commonly used in routine practice (PCR-SSO and SSP), while being very useful for clinical purposes, only capture a part of the molecular information and generate a lot of ambiguities. Hence, genetic studies aiming at comparing human populations are constrained to use frequencies of* HLA* alleles defined nominally at the first and second fields of the* HLA* nomenclature (IMGT/HLA database [15]). These first two sets of digits used to name alleles correspond, respectively, to allele groups and specific protein-coding alleles, both of them being principally defined at the peptide-binding region (PBR) of the* HLA* molecule. Now NGS applied to* HLA* genes will allow taking into account the molecular distances between alleles in terms of nucleotide differences (which were not considered with alleles defined nominally), the molecular variation due to synonymous substitutions in the coding regions (the third set of digits in the* HLA* nomenclature), and the molecular variation due to substitutions in noncoding regions, that is, introns and 3′–5′ untranslated (UTR) regions flanking exons and introns (the fourth set of digits in the* HLA* nomenclature). In this section, we explain how this new material may improve the assessment of population diversity.

### 2.1. Molecular Distances between* HLA* Alleles

*HLA* alleles may differ from each other by one to multiple nucleotide substitutions, the range of which depends on the locus. Taking the whole set of alleles observed in a given population sample and their frequencies, a fine description of the total molecular diversity of this sample can be represented by the distribution of pairwise nucleotide differences among these alleles, named “mismatch distribution.” Mismatch distributions plotted for 56–106 human populations based on* HLA* alleles defined at the second field of the* HLA* nomenclature [21] for different loci revealed different shapes between some populations (the meaning of which is discussed below) but also very large variances; this indicates that in addition to high numbers of alleles found at different frequencies all human populations carry sets of alleles that exhibit a very large spectrum of molecular distances between them.

**Figure 1:**
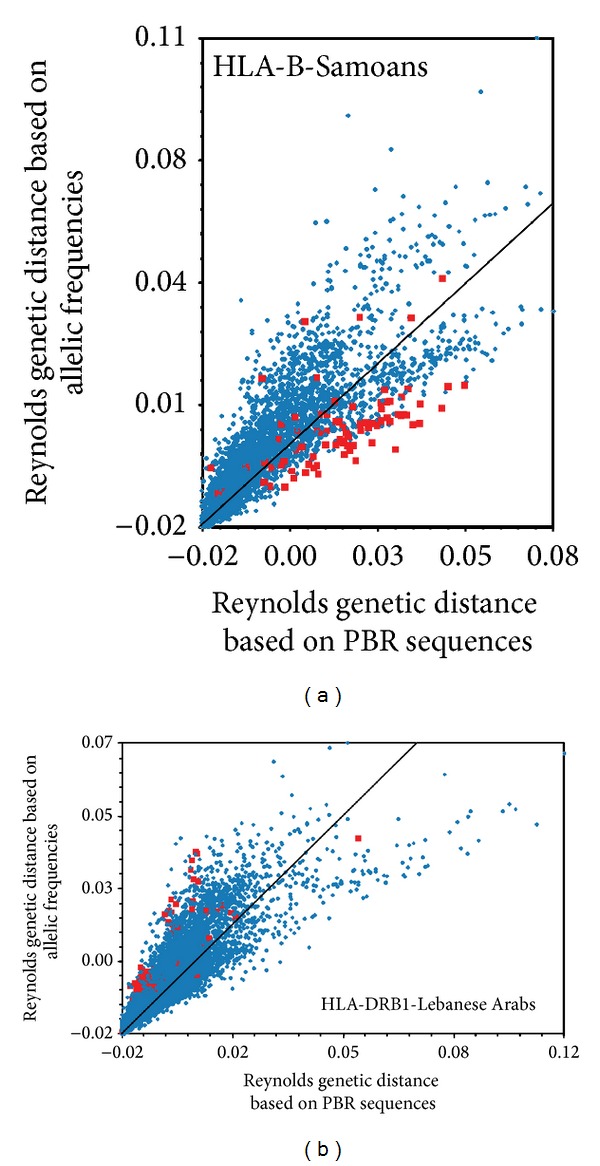
Plot comparisons of two different measures of genetic distances among populations. Each point corresponds to a pair of populations (taken from a large database of 90 populations typed for* HLA*-B and 106 populations typed for* HLA*-DRB1, resp.) for which two values are reported: the genetic distance estimated by taking into account both the allele frequencies and the molecular distance between the alleles (“Φ_st_-derived” Reynold's genetic distance based on PBR sequences, *X* axis) and the genetic distance estimated only from allele frequencies (“*F*_st_-derived” Reynolds' genetic distance based on allele frequencies, *Y* axis). (a) Locus* HLA*-B; in this plot, the population pairs formed by the Samoans and all other populations are highlighted by red squares. (b)* HLA*-DRB1; in this plot, the population pairs formed by the Lebanese Arabs and all other populations are highlighted by red squares. For Samoans at* HLA*-B, genetic distances are clearly skewed toward higher values with Reynold's genetic distance based on PBR sequences, while the opposite situation is observed for the Lebanese Arabs at* HLA*-DRB1. Taking into account the molecular information has thus substantial effects on the estimation of population relationships (from [21]).

Interestingly, this molecular information can be taken into account to compute genetic distances among populations. For example, the commonly used Reynold's genetic distances [26], which is usually derived from a *F*_st_ index [27] based on allele frequencies, can also be computed from a Φ_st_ index [28] based on both allele frequencies and the molecular distances between alleles. As illustrated in Figure 1, Φ_st_-derived distances may deviate to a large extent from more classical *F*_st_-derived genetic distances, suggesting that the introduction of* HLA* sequence information (even though limited to nonsynonymous substitutions in the given example) may substantially change our assessment of population relationships.

### 2.2. Synonymous and Nonsynonymous Substitutions

Taking into account synonymous substitutions (the third field of the* HLA* nomenclature) may provide even more detailed estimates of genetic relatedness between populations, despite the fact that they are of limited interest for transplantation. The proportion of nonsynonymous is known to exceed that of synonymous substitutions in the PBR (providing evidence of balancing selection) [29–31], but the number of known synonymous substitutions is also becoming quite substantial. Although the number of alleles defined by such silent mutations (i.e., the third field alleles) is still limited (Table 1), many of them are detected in population samples typed by high-throughput sequencing (e.g., [32]). Actually, this may even change to a large extent the overall* HLA* allele repertoire: among the 2127 new* HLA*-A, -B, and -C alleles recently discovered by SBT sequencing in a large set of potential haematopoietic stem cell donors from Germany, United States, and Poland [33], as many as 28–30% of them were defined by synonymous substitutions. This suggests that a significant portion of silent* HLA* polymorphism, so far neglected in population genetics studies, may be relevant to infer geographic and/or historical relationships between populations. A special interest, here, is that synonymous substitutions are more prone to evolve neutrally than nonsynonymous substitutions, in particular when the latter occur in functionally important regions of the molecule like the PBR. Unless hitchhiked by neighboring polymorphic sites strongly submitted to selection (which may often happen, of course), the frequency of such (*a priori*) neutral polymorphisms fluctuates by random genetic drift and other mechanisms related to the demography of populations, such as gene flow and long-range migration. The determination of synonymous variants in the* HLA* region may thus be very informative to study this polymorphism in relation to the history of human populations.

### 2.3. Variation outside the Exons Coding for the PBR

As mentioned above, so far the* HLA* data used in population genetics studies did not take into account molecular variation outside the exons coding for the PBR, that is, other exons as well as introns and 3′–5′ UTR regions flanking exons and introns (the fourth field of the* HLA* nomenclature), except in some recent studies [34]. Although a huge part of the* HLA* molecular variation is concentrated in the PBR-coding exons, polymorphic sites are however distributed all along the 4 Mb* HLA* super-locus [35], and other exons as well as introns sometimes reveal important variation (e.g., 8 intronic substitutions and variation in exon 1 distinguish A∗74 : 01 and A∗74 : 02 : 01, [36]). High-throughput* HLA* sequencing, if applied systematically to population samples from different geographic locations, is thus expected to reveal a large amount of polymorphism outside the traditionally tested exons 2 and 3. The main interest is that many of these sites may not behave as neutral polymorphisms. Indeed, by contrast with the “junk-DNA” theory which has prevailed during the last decades, recent studies suggest that a very large part of regions previously thought to be in the “junk” category do in fact have functional roles (see [37], for a review). Many SNPs located in noncoding portions of the* HLA* region might therefore be associated with regulatory functions, as discussed in more detail below. In addition, due to the high level of linkage disequilibrium observed across the human MHC [38], background selection (i.e., strong negative selection against deleterious mutations) or selective sweep (i.e., strong positive selection in favor of beneficial mutations) may affect the evolution of neutral polymorphic sites located at the vicinity of selected mutations (this is also true for synonymous substitutions in the coding regions, as mentioned above). In this case, the variability of such putatively neutral sites would not mirror the effects of population demography. The great advantages of working with* HLA* sequences is that it allows addressing these issues with more accuracy than in the past: first, by testing deviation from neutrality using methods adapted to DNA sequences (e.g., [39, 40]) for different portions of the MHC region located either in exons, in introns, or in 3′–5′ UTR regions and second, by testing linkage disequilibrium across these regions, thanks to the putatively nonambiguous reconstruction of haplotype blocks. These approaches may provide a qualitative assessment, in terms of evolutionary significance, of the molecular variation observed in different* HLA* region, which may then serve to determine the most appropriate region to retain for further population genetics studies.

## 3. Inferring Population Historical Relationships from* HLA* Sequence Data

From a molecular point of view,* HLA* sequencing is expected to generate very precise information on human genetic diversity (as explained above) and to provide an abundant source of information to infer human history. In this section, we first discuss potential problems and pitfalls linked to the generation of this new kind of data, and we then examine some attractive approaches to deal with them.

### 3.1. The Problem of Multiplying the Number of* HLA* Alleles

By taking into account new potential sources of DNA variability both within (with synonymous mutations) and outside (with other exons and with introns and 3′–5′ UTR) the PBR exons, high-throughput* HLA* sequencing is expected to increase very fast the number of observable* HLA* alleles. So far, the number of common and well-defined alleles at classical* HLA* loci has already increased more than threefold between the first two versions of the ASHI catalogue [41, 42]. Moreover, catalogues based on different population sets do not overlap completely [43, 44], which predict major allelic booms when new populations from all over the world are genotyped. The generalization of NGS to* HLA* will probably reveal new common alleles within some known highly frequent two-field (or three-field) allele groups with distinctive haplotypic associations (e.g., DQA1∗01 : 01 or DQA1∗01 : 02), which will definitely lead us to revise both our records of disease-associated alleles or haplotypes and our expectations regarding donor-recipient matching in tissue transplantation. However, many alleles are also expected to be observed as unique sequences, not only because new variants are likely to be constantly generated by gene conversion or point mutations and lost by purifying selection or genetic drift [45], but also because the chances of detecting identical rare alleles in population samples of limited size (which is generally the case in real studies, to the exception of donor registries) are low. We are thus probably reaching a situation where allele frequencies, being too small to be accurately estimated if sample sizes are not massively increased, become unusable in many population data analyses like those involving the computation of genetic distances among populations (as already occurs with* HLA* haplotype frequencies). Also, more qualitative measures of population relatedness like allele sharing, although very attractive [46], are hardly applicable in current sampling conditions. Unless extensively defined* HLA* alleles are recoded to lower levels of resolution, other approaches have thus to be adopted.

Interestingly, this pattern reflects a feature that has been observed in genome-wide sequencing studies: as sequence data probes genetic variation at higher coverage and for larger samples, the proportion of genetic variants which are very rare (i.e., restricted to one or very few individuals) becomes increasingly prevalent. For example, in the exome survey of the 1000 genomes data, which generated high coverage sequencing of coding regions, the number of unique variants increased largely with respect to that seen in previous surveys [47]. This suggests that a similar trend will be observed for* HLA* data, with many novel rare variants being discovered as sequencing surveys expand. Rare variants play a key role in contemporary population genetics, since they contribute substantially to the overall pool, but individually each variant is rare, making a study of its evolutionary and functional impacts challenging [48].

### 3.2. The Pitfall of Gene Trees

In a situation where genetic diversity is described by many single sequences, one temptation is the reconstruction of phylogenetic trees, which represent putative evolutionary relationships between alleles from their sequence homology. This approach was successfully applied to human and chimpanzee MHC sequences as it led to the conclusion that most* HLA* lineages predate the divergence of these two species some 7-8 million years ago ([49], recently confirmed by [50]), while* HLA* alleles are much more recent [51]. This long persistence time of* HLA* lineages was explained by balancing selection [49, 52, 53]. When population genetic studies focusing on human evolution switched from “classical markers” (blood groups, allozymes, etc.) to DNA-typed polymorphisms, inferences on population history, including the famous Out-of-Africa hypothesis [54], were also based on phylogenetic trees.

Actually, these approaches introduced a lot of confusion. First, gene trees are easier to build than to interpret. Not only can different molecular distances and algorithms be used to build a tree, but the number of possible trees for a given set of DNA sequences is excessively large, with the consequence that the retained final tree is not necessarily the most robust. To infer the time to the most recent common ancestor (i.e., the age) of a given allele lineage or of the entire tree also supposes a “molecular clock,” that is, a constant mutation rate across successive generations, which is hardly verified for short evolutionary times, and even less when complex evolutionary mechanisms (like the shuffling of* HLA* genes by gene conversion) are at work. In addition, the calibration of the molecular clock needs information on well-documented fossil remains, which most often does not exist. But besides all these complications, the principal pitfall is actually to believe that gene trees reproduce population trees, when it is not the case [55, 56]. First, each gene has its own genealogy, generally different from the genealogy of populations; secondly, genes circulate horizontally across populations through gene flow, meaning that genetically close populations may have a geographic, rather than a historical relationship; thirdly, the age of a given lineage has nothing to do with the age of a population, genes being generally much older. Putative evolutionary relationships between* HLA* alleles may sometimes be relevant for population migrations' history; an example is given by [57], who relates the presence of DRB1∗08 : 02 : 01 in East Asians and the same allele plus several of its subtypes in Amerindians to “the founder migrations from Asia to America through the Bering Strait.” However, while this example reminds us that rare alleles may be shared by populations located in geographically distant regions and may hence represent a possible legacy of their common ancestry, such interpretations should be taken with caution when based on observations done on single genes.

### 3.3. Understanding Diversity through the Coalescent Approach

**Figure 2:**
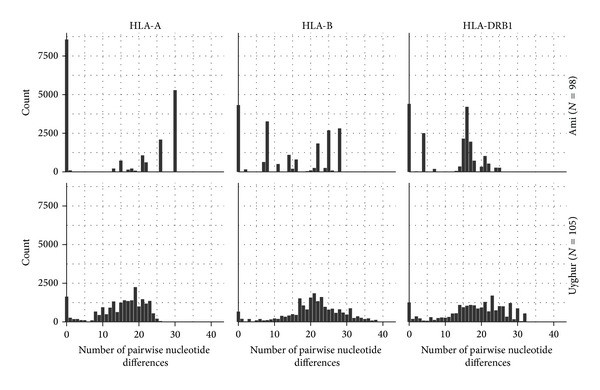
Distributions of the number of nucleotide differences between pairs of sequences (mismatch distributions) in two Asiatic populations of close sample sizes, one Taiwanese (Ami, *N* = 98, top) and one continental (Uyghur, *N* = 105, bottom), based on DNA sequences of* HLA*-A,* HLA*-B, and* HLA*-DRB1 alleles defined at high resolution (sequences downloaded from IMGT/HLA database). While the distributions exhibit a likely unimodal shape for the Uyghur, suggesting recent demographic expansion, they are very irregular for the Ami, reflecting population stationarity or contraction (note that the zero class corresponds to identical sequences, reflecting a high proportion of homozygosity). Besides these demographic signatures, Tajima's *D* are positive for all distributions, in agreement with balancing selection acting on these loci (not shown).

A main alternative to the phylogenetic approach is to use coalescent theory [58], which analyzes the relationships between the molecular diversity of a sample of observed DNA sequences and their possible genealogy (i.e., the history of their inheritance across generations, not to be confounded with a phylogeny). As defined above, the molecular diversity of a sample of observed DNA sequences can be described by a “mismatch distribution.” The latter is expected to be multimodal for populations at demographic equilibrium or having passed through a recent bottleneck, and unimodal in case of either demographic or range expansion (i.e., large spatial dispersal) with high levels of migration between neighboring populations [59–61].  Figure 2 shows an example where mismatch distributions have been obtained at the* HLA*-A, -B, and -DRB1 loci for two population samples of comparable sizes, Central Asian Uyghur and Taiwanese Ami. Although, in these examples, synonymous mutations are not taken into account (which underestimates the total diversity), we note that the shapes of these distributions markedly differ between the two populations; they are compatible (at the 3 loci) with demographic signals of demographic or range expansion, for the Uyghur, and with population contraction, for the Ami, suggesting distinct (and plausible) demographic histories of these two populations.

However, in molecular evolution different mechanisms linked either to demographic history or natural selection sometimes generate similar genetic signals and are thus difficult to disentangle. This has already been stressed for* HLA* [21, 23]. In the case of mismatch distributions, selective sweep or purifying selection may mimic the effects of demographic expansion by generating unimodal shapes (due to an accumulation of low frequency alleles); on the contrary, balancing selection may mimic the effect of population contraction by generating multimodal profiles (due to the maintenance of intermediate frequency alleles). A commonly used statistic to assess the significance of the selection signal is Tajima's *D* [40], which is expected to be negative, in the first case, and positive, in the second case. Tajima's *D* values estimated on the examples given above are all positive and significant (in both populations and at all loci, but to be confirmed when synonymous substitutions are also taken into account). This agrees* a priori* with an effect of balancing selection at all loci and in both populations. However, this balancing selection would not have been strong enough to erase the signals of demography, as suggested by the very distinct shapes of the mismatch distributions obtained for the Uyghur and the Ami. Although the interpretation of mismatch profiles may be more challenging in case of high rates of recombination or gene conversion, this example emphasizes the interest of using* HLA* sequences in a coalescent approach.

### 3.4. Taking into Account Linkage Disequilibrium and Variation at Noncoding Regions

*HLA* sequencing can be used, along with other analytical tools, to infer phased data (either at the level of SNPs within a locus or among loci), allowing us to better estimate linkage disequilibrium. The assessment of linkage disequilibrium as a whole is most useful to infer population history and demography (see, e.g., [62]). Indeed, LD is expected to decrease with time through recombination and thus to be low in populations of remote ancestry; in agreement with this prediction, lower LD has been found in an African population compared to a European, a Chinese, and a Japanese population [63] based on a SNP haplotype map of the extended* HLA* region (7,5 Mb). This is congruent with genome-wide studies (e.g., [64]). On the other hand, LD is mainly generated by genetic drift which occurs very fast in small and isolated populations; such populations are therefore more prone to exhibit regions in strong LD.

Although being functionally important and hence likely to be submitted to significant selection, DNA variability at noncoding regulatory regions might also be informative for the reconstruction of human evolution. Indeed, population differences in these regions could correspond to signatures of adaptations to peculiar environments during human evolution, for example, characterized by distinct pathogen richness or prevalence, climates, food resources, and others. These kinds of associations might be assessed through functional studies and an interdisciplinary approach involving very distinct domains such as epidemiology and archaeology.

### 3.5. Reconstructing Human Peopling History through Computer Simulations

As several evolutionary forces govern simultaneously the evolution of the* HLA* polymorphism, demographic events can be inferred from DNA sequence data as long as one also takes into account the effect of selection, and vice versa. Several methods have been developed in this perspective (e.g., [65, 66]), but one of the most widely used today is computer simulation of plausible scenarios for human peopling history coupled with approaches allowing the estimation of several kinds of parameters at the same time (e.g., [67]). Two studies have already applied this approach to* HLA* data to explore different scenarios of peopling history, that is, for the Western Mediterranean region [22] and for East Asia [68]. Both demographic parameters (population density, demographic growth, and migration) and balancing selection coefficients on several* HLA* loci have been estimated, leading to relative selection intensities consistent with previous studies [69, 70]. Using this kind of methods on* HLA* sequence (instead of allele frequency) data is even more promising, as it provides estimates for additional parameters, in particular those related to molecular evolution like molecular diversity, mutation rates, and linkage disequilibrium (M. Currat, personal communication, 2014).* HLA* sequencing thus opens many new ways of exploring population variability in the context of both human peopling history and molecular evolution.

## 4. Using* HLA* Molecular Variation to Assess Regulatory Functions and Diseases

The exponential increase of genomic data has deeply influenced human genetics and has also influenced the way genetic variation in* HLA* genes is analyzed and interpreted. In this section, we discuss how the use of genotyping arrays and whole genome sequencing have been incorporated into the analysis of* HLA* variation. We also address the challenges in using next generation sequencing to make inferences about the expression of* HLA* genes.

### 4.1. SNPs in the* HLA* Region and GWAS Studies

Over the last decade, the development of technologies allowing the genotyping of thousands of markers throughout the genome has bolstered the implementation of genome-wide association studies (GWAS), which are designed to search the genome for genetic variants whose presence is statistically correlated with a disease phenotype. The scale of GWAS has grown dramatically, both in terms of genome coverage and sample sizes, and it has become possible to implement investigations with up to thousands of samples surveyed in over millions of SNPs (e.g., Welcome Trust Case Control Consortium, 2007). The large sample sizes play an important role, given that there is relatively low power to detect genetic variants that contribute to disease risk. The large number of SNPs is likewise important, since it allows researchers to narrow down the region which contains variants associated with the phenotype of interest.

Various GWAS have identified SNPs that lie within the MHC region, or within* HLA* genes themselves, as being statistically associated with disease phenotypes. Overall, whereas the MHC region makes up only 0.3% of the genome, a catalog of genome-wide significant SNPs identifies that 6.4% are in the MHC [71], showing a marked overrepresentation of variants contributing to the studied phenotypes in this region.

The range of disease phenotypes that show associations with genetic variants within the MHC is vast (reviewed in [13]), spanning autoimmune disorders (e.g., type 1 diabetes, rheumatoid arthritis, and psoriasis), response to infections (e.g., outcome of infection by dengue, HTLV-1, HIV), neuropsychiatric disorders (e.g., narcolepsy, schizophrenia), and specific forms of cancer (e.g., Hodgkin's lymphoma).

The finding of an association between a SNP and a disease phenotype raises important challenges. First, it is desirable to establish whether that genetic variant is tagging a polymorphism that contributes to the phenotype, or whether it is causally related to the phenotype. Secondly, the finding of an initial strong association in a GWAS may make the identification of subsequent weaker yet statistically and biologically relevant associations harder to find, calling for methods which sequentially remove detected associations to facilitate the identification of weaker ones. In the case of associations of SNPs within the MHC region, these challenges are particularly relevant given the extensive LD and high gene density, implying that causal and noncausal variants are likely to cosegregate (i.e., be in complete or very high LD) and that multiple genetic variants contributing to a disease phenotype may be localized close to one another, making it challenging to unravel their specific roles.

Analyzing variation in the MHC in the context of genome-wide association studies has allowed some of these challenges to be tackled. First, the large number of SNPs allows additional targets within the MHC region to be identified, often revealing loci that were not postulated as candidates. Secondly, the simultaneous analysis of SNPs in the MHC region and elsewhere allows the effect sizes (a measure of the degree to which genetic variation at that locus explains variation in the phenotype of interest) to be estimated. In this way, the relative contribution of genetic variation at* HLA* and non-*HLA* loci can be compared. Such approaches have shown cases where* HLA* genes explain the vast majority of phenotypic variation (e.g., ankylosing spondylitis) as well as those where non-*HLA* contributions can involve several loci (e.g., rheumatoid arthritis).

Interestingly, the identification of GWAS markers which are not in the MHC has required performing novel rounds of statistical testing after SNPs from the MHC were removed or thinned out (i.e., a single SNP with strong association is kept), since the presence of multiple strongly associated SNPS in the MHC limits the ability to detect additional associations.

### 4.2. Using SNPs to Infer* HLA* Genotypes

Genetic data on human populations can currently be generated by both genotyping with microarrays, or by whole genome sequencing. Although techniques of NGS are becoming increasingly cheaper, microarrays are still more cost effective on very large samples, and most studies with very large samples are based on SNP genotyping [72]. Over the last years, a powerful technique called* genotype imputation* has been developed, which allows the information from NGS studies to be used to boost the informativeness of SNP genotyping surveys. Imputation can be defined as the use of information on haplotype variation in a reference sample, typically generated by DNA sequencing, to make predictions of genotypes in a study sample, which was genotyped at only a subset of these SNPs. Imputation approaches effectively allow DNA sequence data to be obtained using statistical approach and have been shown to increase the power of GWAS [73] by increasing the number of variants available.

Recently, several research groups have proposed that imputation methods can be extended to the* HLA* genes. The logic here is the same as for genome-wide imputation studies: prior knowledge on the association between SNPs in a genomic region (in this case, the MHC) and specific haplotypes (in this case, the* HLA* alleles present) allows* HLA* genotypes to be inferred for individuals that were not typed or sequenced at these loci but that have genome-wide SNP data (which encompasses the MHC region). In the context of the theme of the present review, these* HLA* imputation methods can be seen as a way to tap into the rich resource of contemporary genomic data and generate information which approximates that of direct NGS of* HLA* genes, but relying on an inferential approach. Although not directly comparable to the direct sequencing of* HLA* alleles, these imputation approaches do enhance the power of association studies in the MHC region as will be discussed below.

One class of* HLA* imputation approaches simply extends the logic of SNP tagging to the context of* HLA* genes (e.g., [63]), but the extremely high polymorphism of* HLA* genes results in an inefficient performance. A second approach consists in using the haplotype structure of the SNPs surrounding an* HLA* locus to make a prediction of what* HLA* alleles an individual carries [74, 75]. This latter approach relies on the availability of a training set (i.e., a sample for which* HLA* and SNP data are known and phased), as well as phased data for the SNPs which will be used to infer the* HLA* allele. Another approach circumvents the need for* a priori* haplotype information concerning* HLA* and SNPs and is implemented in HIBAG [76].

These methods have shown some promising results, with imputation accuracies above 90% for common second field level alleles in samples of Europeans typed for a set of 191 SNPs in the MHC region [76]. However, such a high imputation success has also been challenged [77].

The possibility of using large datasets for which SNP genotypes are available to infer the* HLA* alleles is attractive on several grounds. First, it allows existing genetic data, often generated without the goal of characterizing* HLA* variation, to be used to gain a better understanding of these genes. Secondly, it implicitly allows the imputation of genetic variation at a high molecular level of resolution. For example, the inference of a second-field level* HLA* allele implies that amino acid residues at specific positions can be inferred and have their effects tested upon phenotypes of interest. Because there is* a priori* evidence that the variation at specific amino acids can contribute in a statistically significant way to disease phenotypes, obtaining data at this level of resolution is an important methodological resource as will be discussed in the next section.

Although imputation approaches may be powerful in inferring the frequencies of common variants, critical to GWAS studies, they are not as useful in uncovering the contributions of rare variants, which by definition are difficult to impute, given their low frequency in reference panels. For these, direct sequencing of the* HLA* genes remains the necessary strategy. In this context, it is important to consider challenges associated with* HLA* genotyping in the context of NGS. While specific protocols are being generated for* HLA* genotyping based on NGS (e.g., [78]), it is clear that features of the* HLA* genes make genetic analyses particularly challenging. First, the extensive similarity among paralogous genes results in many reads mapping to multiple loci, a feature that in practice leads them to be discarded by aligners. In addition, when NGS reads are mapped to the reference human genome, many reads are discarded because they show many mismatches with the sequences present in the reference (an expected finding, given the high polymorphism of the region). These two features of sequencing analyses in the* HLA* region imply that conventional analysis pipelines end up discarding a large proportion of reads (due to multiple mappings or to extensive mismatches), resulting in loss of information for SNPs in the* HLA* genes. In line with this, Major et al. [79] have shown that high coverage data is essential in order to make correct inferences about* HLA* genotypes based on NGS. This implies that customized procedures must be implemented in order to efficiently genotype SNPs in the* HLA*.

The analysis of* HLA* genes will benefit greatly from the use of NGS data, since the discovery of variants via NGS will allow imputation-based analyses to take into account an increasingly extensive set of SNPs, including regulatory and intronic variants. The inclusion of these variants into association studies will be an important development, since many play an important role in disease association and can have their effects assessed experimentally (e.g. for regulatory variants).

### 4.3. Dissecting Associations within the MHC Region

For many diseases, candidate gene approaches had previously shown the association of specific* HLA* alleles with a disease phenotype. However, it often remained unclear if the association was causal or if the* HLA* locus was simply tagging a causal variant, and if further genes in the MHC region were associated with the disease phenotype. In addition, the identification of specific amino acids within the* HLA* locus contributing to the disease phenotype remained a challenge, although hypotheses had been raised regarding specific motifs (shared epitopes) whose presence contributed to the disease phenotype.

The availability of large numbers of SNPs and samples has allowed researchers to disentangle the contribution of different markers within the MHC region to search for novel variants and to identify molecular-level variants contributing to the disease phenotype [71, 80].

A good example of how the contribution of multiple loci was disentangled comes from a GWAS for Sjögren's syndrome, which initially detected multiple SNPs within the* HLA* region as associated with the disease [81]. Using logistic regression, a technique that allows the influence of one marker to be accounted when testing for that of another, two independent effects were detected. A further analysis consisted in imputing* HLA* alleles, allowing the GWAS findings to be compared to those of previous candidate gene studies, which focused on* HLA* loci. In the case of Sjögren's syndrome, the* HLA* alleles previously associated with the disease were confirmed, but the GWAS framework allowed SNPs and* HLA* alleles associated with a phenotype to have their effects disentangled, in this case showing that a GWAS SNP was a better marker for the phenotype than the associated* HLA* allele.

The GWAS framework also allows functional hypotheses underlying associations to be probed. One way to do this consists in testing if the set of SNPs which show associations with the disease shows enrichment for some specific genomic feature (e.g., regulation-related motifs, structural variation). In the case of Sjögren's syndrome, significantly associated SNPs showed a large excess of localization in transcription factor binding sites [81].

A particularly fine dissection of the contribution of variants was achieved in a study of rheumatoid arthritis, where imputed* HLA* alleles were used to predict the genotype at specific amino acid positions in a very large sample (5,018 cases and 14,974 controls) [82]. Such a large dataset allowed associations at the molecular level to be tested, and five amino acid positions were detected as independently contributing to disease risk: 3 from DRB1, one from* HLA*-B, and one from DPB1. Such a study exemplifies that large sample sizes, associated with genome-wide typing and imputation of* HLA* alleles, allow the dissection of the genetic basis of a complex disease phenotype, identifying the independent contribution of multiple loci and specific sites within the MHC region.

### 4.4. Unraveling the Expression Patterns of* HLA* Loci

In addition to the coding variation within the gene itself, variation in the expression level of* HLA* genes may also result in important phenotypic differences related to disease status or survival probability. It is therefore of interest to understand how* HLA* expression varies and to gain insight into what are the genetic variants that control it.

Quantifying gene expression has typically been accomplished by using techniques that estimate the abundance of RNA molecules originating from a locus, and microarrays have been employed to this end. In the case of* HLA* genes, however, an important limitation arises because of the high polymorphism of the genes, which may cause transcripts to fail to hybridize to the probe, assuming they span a region that is polymorphic. In light of this, expression data for* HLA* genes obtained from commercial arrays is of limited reliability.

Solutions to these problems have been to filter out results associated with probes that match regions which are found to be polymorphic in population level sequencing studies and to design custom arrays that account for the known polymorphism [83]. Analyses that used these strategies have shed light on important aspects of MHC region expression. First, cell lines homozygous for distinct MHC haplotypes were shown to have marked differences in expression, suggesting that a haplotype-specific expression profile exists. Secondly, genetic variants which are correlated with expression patterns (known as expression QTLs, or eQTLs) were shown to be overrepresented in the MHC region, providing a useful list of putative regulatory variants [83]. The limitations of these studies include the fact that samples do not typically account for geographic variation (with the two cited studies focusing on individuals of European ancestry) and the fact that expression patterns of cell lines homozygous for the MHC may not capture the full spectrum of variation in this genomic region, calling for broader population-based studies.

The analysis of eQTLs has also proved useful in interpreting GWAS results. Nica et al. [84] showed that eQTLs are abundant among GWAS SNPs, consistent with their regulatory role contributing to disease phenotype. This rationale can be extended to the MHC, and Lessard et al. [81] showed that SNPs in the MHC associated with Sjögren's syndrome are frequently eQTLs, again indicating that polymorphisms affecting transcription levels may explain associations. These studies indicate that over the next years we should expect a tighter integration between data on genotypic variation within and around* HLA* genes and that of phenotypic variation, in the form of expression patterns. However, the challenge of developing methods that can estimate* HLA* expression reliably, in particular from RNAseq assays, which are highly challenging to perform in such a polymorphic region, remains to be addressed [85].

## 5. Conclusion

By entering this new genomic era characterized by high throughput sequencing,* HLA* may in some way be regarded as an ideal polymorphism for population geneticists interested by human diversity and evolution. Indeed, a very wide range of issues can be addressed through its fine molecular analysis, from questions related to human peopling history and natural selection to the identification of markers involved in diseases and regulatory functions. However, one should also be aware of the potential loss of statistical power if these extraordinary large amounts of new genetic data are not analyzed with a correspondingly huge increase of sample sizes: then any conclusion drawn from these studies may be as insignificant as any other result occurring by chance.

## Figures and Tables

**Table 1:** Number (#) and proportion (%) of *HLA* alleles defined at the second, third, and fourth fields of the current *HLA* nomenclature (IMGT/HLA database release 3.15.0, 2014-01-17). The third field (highlighted in bold) is defined by synonymous nucleotide substitutions in coding regions.

Locus	Total	Second field		Third field		Fourth field	
	#	#	%	#	%	#	%
A	2579	1862	72.2%	**691**	**26.8%**	26	1.0%
B	3285	2404	73.2%	**858**	**26.1%**	23	0.7%
C	2133	1486	69.7%	**616**	**28.9%**	31	1.5%
DRA	7	0	0.0%	**4**	**57.1%**	3	42.9%
DRB1	1411	972	68.9%	**429**	**30.4%**	10	0.7%
DQA1	51	23	45.1%	**13**	**25.5%**	15	29.4%
DQB1	509	318	62.5%	**181**	**35.6%**	10	2.0%
DPA1	37	15	40.5%	**17**	**45.9%**	5	13.5%
DPB1	248	198	79.8%	**46**	**18.5%**	4	1.6%
